# CMHT GP Fortnightly Consultation Clinics – A Pilot Adjunctive Model for GP Access to Mental Health Advice

**DOI:** 10.1192/bjo.2025.10467

**Published:** 2025-06-20

**Authors:** Mike Apio, Aamenah Hawash

**Affiliations:** 1 Priory Hospital, London, United Kingdom; 2 Luton GP VTS, Luton, United Kingdom

## Abstract

**Aims:** Assessing the referral patterns, patient characteristics, constraints, immediate and wider benefits of a GP/CMHT fortnightly consultation-liaison clinic as a component of an enhanced community mental health service design.

**Methods:** A Transformation initiative pilot GP/CMHT Consultation-Liaison huddles convened over 20 (21) months via video-link; January–December 2021 (23) to September 2022 (14). Initial session in December 2020 enabled both teams comprising two managers, GP, Psychiatrist, Primary care link worker and GP community outreach specialists and CMHT admin support established the format of the Hour long sessions. At various times Other GP Colleagues/Specialist Addiction Services/Sexual Health Consultants/team members/Memory Clinic Specialists joined as appropriate. The sessions provided opportunities for a few video-linked patient consultations and trainee observations. Ahead of the fortnightly sessions is an email list of patients from GP to Psychiatrists with specific queries. Number of patients range from 4 (2) to 12 (14) each session. However, some queries were addressed ahead of sessions or concluded at the meeting. Enquiries varied, ranging from referrals tracking, medication or management advice, diagnosis, risk mitigation strategies and learning on incidents.

**Results:** Total 354 patient encounters were listed or discussed with number of patients per sessions ranging from 2 to 15 mean of (9). Total 37 sessions with 223 patients (2021) and 131 (2022) discussed. Recurring patient encounters range from 2–14 times. Non-recurring patients overall 113 (32%). All patients were within working age group, with the youngest aged 17 plus and the oldest 67 years (four patients). Mean age 38.4 years. Patient characteristics, diagnoses, risks and immigration issues and impact to access to services frequently encountered. Presentations discussed varied with more complex cases frequently recurring.

**Conclusion:** Consultation liaison model has evolved over the years. With recent pandemic, demand for secondary care interventions has increased to the degree innovative approaches offer alternatives to mitigate risks, support primary care services, strengthen GP confidence and most importantly, improve transfer to primary care. Further research is required to strengthen approaches to CMHT/Primary care interfaces.

